# Adipocyte arrestin domain-containing 3 protein (Arrdc3) regulates uncoupling protein 1 (Ucp1) expression in white adipose independently of canonical changes in β-adrenergic receptor signaling

**DOI:** 10.1371/journal.pone.0173823

**Published:** 2017-03-14

**Authors:** Shannon H. Carroll, Ellen Zhang, Bing F. Wang, Katherine B. LeClair, Arifeen Rahman, David E. Cohen, Jorge Plutzky, Parth Patwari, Richard T. Lee

**Affiliations:** 1 Harvard Stem Cell Institute and Department of Stem Cell and Regenerative Biology, Harvard University, Cambridge, Massachusetts, United States of America; 2 Division of Gastroenterology, Hepatology and Endoscopy, Brigham and Women’s Hospital and Harvard Medical School, Boston, Massachusetts, United States of America; 3 Cardiovascular Division, Brigham and Women’s Hospital, Harvard Medical School, Boston, Massachusetts, United States of America; Tohoku University, JAPAN

## Abstract

Adaptive thermogenesis and cold-induced activation of uncoupling protein 1 (Ucp1) in brown adipose tissue in rodents is well-described and attributed to sympathetic activation of β-adrenergic signaling. The arrestin domain containing protein Arrdc3 is a regulator of obesity in mice and also appears linked to obesity in humans. We generated a mouse with conditional deletion of *Arrdc3*, and here we present evidence that genetic ablation of *Arrdc3* specifically in adipocytes results in increased Ucp1 expression in subcutaneous and parametrial adipose tissue. Although this increase in expression did not correspond with significant changes in body weight or energy expenditure, adipocyte-specific *Arrdc3*-null mice had improved glucose tolerance. It was previously hypothesized that Arrdc3 ablation leads to increased β-adrenergic receptor sensitivity; however, *in vitro* experiments show that *Arrdc3*-null adipocytes responded to β-adrenergic receptor agonist with decreased Ucp1 levels. Additionally, canonical β-adrenergic receptor signaling was not different in *Arrdc3*-null adipocytes. These data reveal a role for Arrdc3 in the regulation of Ucp1 expression in adipocytes. However, this adipocyte effect is insufficient to generate the obesity-resistant phenotype of mice with ubiquitous deletion of *Arrdc3*, indicating a likely role for Arrdc3 in cells other than adipocytes.

## Introduction

Uncoupling protein 1 (Ucp1) is a principal driver of adipose thermogenesis in mammals [1,2], yet the regulation of its expression and activity in beige adipocytes is incompletely understood [3]. In brown adipocytes, norepinephrine stimulation of the β2 or β3 adrenergic receptor results in canonical G protein coupled receptor signaling through adenylyl cyclase followed by protein kinase A (PKA) activation, which leads to phosphorylation of cAMP response element binding protein (CREB) and promotes Ucp1 expression [4]. β-adrenergic activation also stimulates lipolysis via PKA phosphorylation of hormone sensitive lipase (HSL), which provides the fatty acids that are necessary for Ucp1 activity [4]. Additionally, Ucp1 levels increase with β-adrenergic stimulation due to decreased turnover of the protein by lysosomal degradation [5]. However this mechanism has not been fully elucidated.

Previously we identified arrestin domain-containing 3 protein (Arrdc3) as a regulator of obesity through the combination of human linkage data, human expression data, and mouse genetic studies [6]. *Arrdc3*-null mice appeared resistant to obesity through increased energy expenditure and increased Ucp1 expression in white adipose tissue [6]. Furthermore, adipose tissue from *Arrdc3*-null mice had increased responsiveness to adrenergic stimulation, as measured by cAMP concentration and lipolysis [6]. Our laboratory and others have reported that Arrdc3 interacts with the β2-adrenergic receptor and may be involved in its recycling [6–11]. Arrdc3 contains a PPXY domain, which is known to bind WW domain-containing proteins, particularly the HECT-domain containing E3 ubiquitin ligase [11]. Qi et al. found Arrdc3 to interact with Nedd4 ubiquitin ligase in overexpressing cells [12]. Most recently it has been shown that Arrdc3 interacts with the β2-adrenergic receptor in the early endosome and prevents receptor recycling [9]. As the β2-adrenergic receptor continues to signal from the endosome [13,14] it is presumed that this delayed residency affects adrenergic signaling. The subsequent cellular consequences of the interaction of Arrdc3 with ubiquitin ligase and with the β2-adrenergic receptor remains to be tested directly.

In both the mouse and human, Arrdc3 is widely expressed, and its expression is regulated by feeding/fasting in several metabolically relevant tissues [6]. The signal for Ucp1 activation and thermogenesis can begin with signaling via the central nervous system, release of norepinephrine from sympathetic neurons and activation of the β2-adrenergic receptor on adipocytes [15]. Therefore it is unknown whether the increased browning of white adipose tissue in the mouse with ubiquitous deletion of *Arrdc3* is a consequence of *Arrdc3* deletion in the central nervous system or in the adipocyte, or an alternative cell type. Here we describe an adipocyte-specific *Arrdc3*-null mouse that we generated in order to address two questions; whether deletion of *Arrdc3* in adipocytes recapitulates the total *Arrdc3*-null phenotype and whether deletion of *Arrdc3* in adipocytes results in increased β-adrenergic signaling. Our results reveal that the adipocyte-specific *Arrdc3*-null mouse has significantly increased Ucp1 expression in white adipose tissue; however, this is not associated with significant differences in body weight or energy expenditure. Additionally *Arrdc3*-null adipocytes *in vitro* do not have increased canonical β-adrenergic receptor signaling or increased sensitivity to β-adrenergic stimulation. Therefore, adipocyte Arrdc3 may participate in the regulation of Ucp1 expression in a manner independent of β-adrenergic receptor signaling. Finally, deletion of *Arrdc3* from adipocytes alone was insufficient to cause the energetic differences noted in the total *Arrdc3*-null mouse, indicating a role for Arrdc3 in other tissues as well.

## Results

### Body composition of the adipocyte-specific Arrdc3-null mouse

**Fig 1:**
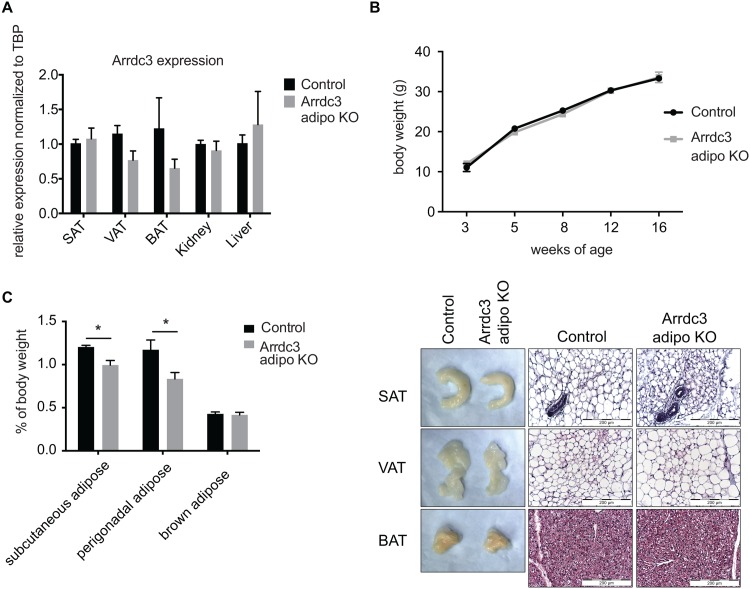
Characterization of adipocyte-specific *Arrdc3*-null mice. (A) To confirm adipocyte-specific deletion, Arrdc3 expression was measured in various tissues of Cre–(control) and Cre+ (*Arrdc3*-null) mice by quantitative PCR. Brown (BAT), parametrial (VAT) and subcutaneous adipose tissue (SAT) had significantly decreased *Arrdc3* expression while there was no significant difference in liver or kidney (n = 3–4). (B) Adipocyte-specific *Arrdc3*-null mice and littermate controls were weighed for 16 weeks and no differences in body weight were found (n = 4–10). (C) Specific adipose depots of female mice were weighed and normalized to total body weight. Subcutaneous (SAT) and parametrial (VAT) adipose tissue from adipocyte-specific *Arrdc3*-null mice weighed significantly less than controls (n = 5). (D) Representative macroscopic (formaldehyde fixed tissue) and microscopic appearance of subcutaneous (SAT), parametrial (VAT) and brown (BAT) adipose tissue from adipocyte-specific *Arrdc3*-null and control mice. Paraffin tissue sections were stained with hematoxylin and eosin and images were taken at 40x.

The mouse with ubiquitous deletion of *Arrdc3* is smaller, gains less weight and has less total adipose as compared to controls [6]. To determine whether the loss of *Arrdc3* in the adipocyte contributes to the total *Arrdc3*-null phenotype, we generated adipocyte-specific *Arrdc3*-null mice. Gene expression of *Arrdc3* in adipose tissue was reduced as expected by quantitative PCR (Fig 1A). Adipocyte-specific *Arrdc3*-null females did not have significant differences in body weight (Fig 1B) and had no significant difference in percent total fat (19.5% vs. 16.6% ± 2.0), as measured by MRI. Tibia length was not different between control and adipocyte-specific *Arrdc3*-null mice (16.3 mm ± 0.2 vs. 16.2 mm ± 0.1). However, when specific adipose depots were isolated, weighed and normalized to body weight, we found that the female adipocyte-specific *Arrdc3*-null mice had significantly less subcutaneous and parametrial adipose tissue, with no difference in brown adipose weight (Fig 1C and 1D). Histological examination of the tissue did not show any obvious differences in cell composition or morphology in the adipocyte-specific *Arrdc3*-null mice relative to controls (Fig 1D).

### Beige adipocyte-associated gene expression

**Fig 2:**
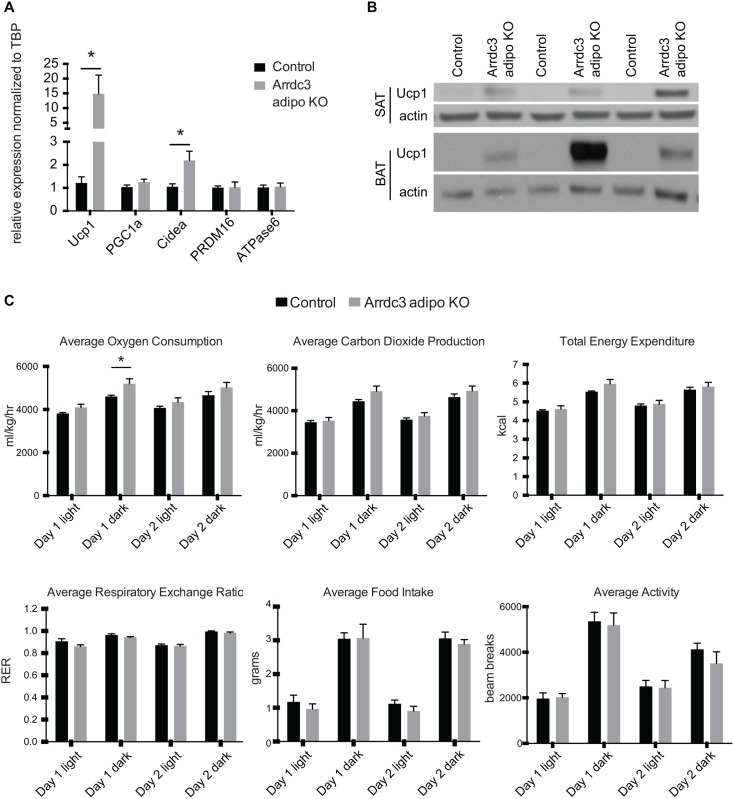
Increased expression of Ucp1 in white adipose tissue of adipocyte-specific *Arrdc3*-null mice. (A) Quantitative PCR analysis of gene expression in subcutaneous adipose tissue (n = 5–9). (B) Western analysis of Ucp1 protein expression in subcutaneous (SAT) and parametrial (VAT) adipose tissue. (C) 48 hours of CLAMS analysis of adipocyte-specific *Arrdc3*-null and control mice at 28°C ambient temperature (n = 5). *p≤ 0.05.

Ubiquitous *Arrdc3*-null mice were previously described to have increased Ucp1 mRNA expression in subcutaneous adipose depots [6], suggesting increased browning of the white adipose tissue. Here, we found that adipocyte-specific *Arrdc3*-null female mice had significantly increased mRNA expression of Ucp1 and moderately increased expression of Cidea in the subcutaneous adipose tissue (Fig 2A). No differences were found in the mRNA expression of the other beige adipocyte makers PRDM16 or PGC1α and there was no difference in the expression of the mitochondrial protein ATP synthase 6 (Fig 2A). Importantly, Ucp1 protein expression was also substantially higher in the subcutaneous adipose of the adipocyte-specific *Arrdc3*-null mice relative to controls (Fig 2B). Whereas there was no significant difference in the mRNA expression of the beige adipocyte genes in the visceral (parametrial) adipose tissue (data not shown), Ucp1 protein expression was highly upregulated in the parametrial adipose tissue of adipocyte-specific *Arrdc3*-null white adipose depot (Fig 2B). Notably, Ucp1 protein expression was significantly higher in the parametrial adipose of adipocyte-specific *Arrdc3*-null mice as compared to expression in the subcutaneous adipose tissue (S1 Fig). Male adipocyte-specific *Arrdc3*-null mice also had no difference in body weights (S2 Fig). Similar to the females, adipocyte-specific *Arrdc3*-null males had significantly higher mRNA and protein expression of Ucp1 in subcutaneous adipose tissue (S2 Fig) but both control and adipocyte-specific *Arrdc3*-null male mice had very little expression in the visceral (epidydimal) depot (data not shown). There was no difference in Ucp1 mRNA expression or protein expression in the brown adipose tissue of adipocyte-specific *Arrdc3*-null mice (data not shown). As male and female adipocyte-specific *Arrdc3*-null mice had similar phenotypes and as we had previously measured the effect of *Arrdc3* ablation on energy expenditure in female mice, we chose to continue our investigation in female mice so that we could reference back to the total null mouse.

As increased Ucp1 expression is often associated with an increase in heat production and energy expenditure [2], we tested these parameters in female control and adipocyte-specific *Arrdc3*-null mice using the comprehensive lab animal monitoring system (CLAMS). There were no differences in food intake or activity between the control and adipocyte-specific *Arrdc3*-null mice (Fig 2C). Adipocyte-specific *Arrdc3*-null mice had a small increase in oxygen consumption during the dark period but this was only statistically significant on the first day of measurement (Fig 2C). Similarly, carbon dioxide production and energy expenditure tended to be higher in the adipocyte-specific *Arrdc3*-null mice on the first day of measurement. Respiratory exchange ratio (RER) tended to be lower in the adipocyte-specific *Arrdc3*-null mice but only during the first day of measurement (Fig 2C).

**Fig 3:**
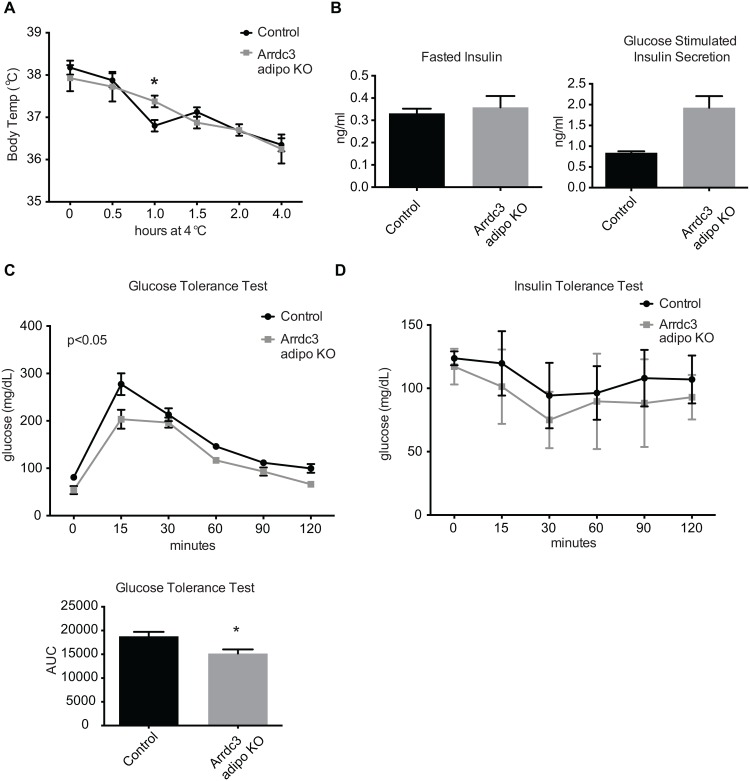
Effect of adipocyte-specific deletion of *Arrdc3* on body temperature and glucose homeostasis. (A) Control and adipocyte-specific *Arrdc3*-null mice were exposed to 4°C for four hours and core body temperature was measured at indicated time points (n = 4). (B) Blood insulin levels of control and adipocyte-specific *Arrdc3*-null mice after an overnight fast and after 2 minutes of i.p. glucose administration (n = 3). (C) Glucose tolerance testing of control and adipocyte-specific *Arrdc3*-null mice (n = 3). (D) Insulin tolerance testing of control and adipocyte-specific *Arrdc3*-null mice (n = 3). *p<0.05.

Increased Ucp1 expression is often correlated with non-shivering thermogenesis and an improved maintenance of body temperature during cold exposure (reviewed in [16]). Total *Arrdc3*-null mice have improved body temperature maintenance after four hours of exposure to 4°C [6]. To determine whether our adipocyte-specific *Arrdc3*-null mice also had improved protection of body temperature upon cold exposure, we exposed female mice to 4°C for four hours. Overall, control and adipocyte-specific *Arrdc3*-null mice had similar changes in body temperature. Only at one hour of exposure did the adipocyte-specific *Arrdc3*-null mice have significantly higher body temperatures (Fig 3A). Increased Ucp1 expression is also often associated with increased glucose tolerance and insulin sensitivity, and total *Arrdc3*-null mice have improved glucose homeostasis [6]. Female adipocyte-specific *Arrdc3*-null mice had no difference in fasted blood insulin levels but had a significantly higher glucose-stimulated insulin response (Fig 3B). Adipocyte-specific *Arrdc3*-null mice had lower blood glucose after fasting overnight (p<0.05 by t-test) and maintained lower glucose concentrations during the course of the glucose tolerance test (Fig 3C). Glucose clearance upon insulin administration in the adipocyte-specific *Arrdc3*-null mice was not different as compared to controls (Fig 3D).

**Fig 4:**
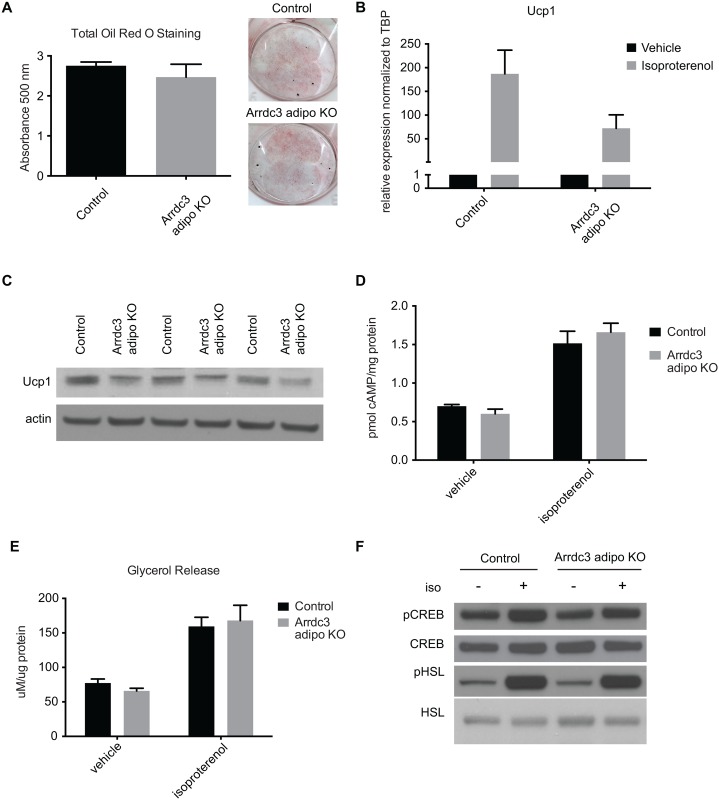
β-adrenergic signaling in *Arrdc3*-null adipocytes *in vitro*. (A) Quantification of total Oil Red O staining of control and adipocyte-specific *Arrdc3*-null stromal vascular fractions after adipogenic treatment (n = 4 mice/group). Representative Oil Red O staining of stromal vascular fraction-derived adipocytes from control and adipocyte-specific *Arrdc3*-null mice. (B) Quantitative PCR analysis of Ucp1 upregulation upon 4 hours of isoproterenol treatment of control and *Arrdc3*-null cells (n = 4 mice/group). (C) Western analysis of Ucp1 protein expression upon 24 hours of isoproterenol treatment in control and *Arrdc3*-null cells. (D) cAMP concentration in control and *Arrdc3*-null cells upon 5 minutes of isoproterenol treatment (n = 3 mice/group). (E) Glycerol concentrations of control and *Arrdc3*-null cell media after 3 hours of isoproterenol treatment (n = 4 mice/group). (F) Western analysis of CREB and HSL phosphorylation of control and *Arrdc3*-null cells upon 5 minutes of isoproterenol treatment (n = 3 mice/group).

We previously reported that whole animal deletion of *Arrdc3* resulted in increased browning of white adipose tissue. *Ex vivo* analysis of control and *Arrdc3*-null whole adipose tissue demonstrated an increase in lipolysis and cAMP levels in response to β-adrenergic receptor agonism [6]. Here we tested the effect of *Arrdc3* ablation on β-adrenergic receptor agonism specifically in adipocytes *in vitro*. Stromal vascular fractions from adipocyte-specific *Arrdc3*-null mice tended to have decreased Arrdc3 expression after adipogenic treatment (S3 Fig) and showed no significant difference in adipocyte differentiation (Fig 4A). Adipocytes derived from the stromal vascular fraction of adipocyte-specific *Arrdc3*-null mice had no significant difference in basal Ucp1 mRNA expression but tended to have decreased induction of Ucp1 mRNA upon isoproterenol treatment relative to control adipocytes (Fig 4B). Additionally, Ucp1 protein expression after isoproterenol treatment was lower in the *Arrdc3*-null adipocytes relative to controls (Fig 4C). Ucp1 mRNA and protein expression were similarly decreased relative to controls when cells were treated with the β3-adrenergic receptor specific agonist CL316243 and with the β2-adrenergic receptor specific agonist fenoterol (data not shown).

To determine whether the differences in Ucp1 upregulation upon isoproterenol treatment in *Arrdc3*-null adipocytes corresponded to differences in β-adrenergic signaling, we measured components of the β-adrenergic receptor signaling cascade. *Arrdc3*-null adipocytes had similar levels of cAMP (Fig 4C) and a similar lipolytic response (Fig 4D). Further, *Arrdc3*-null adipocytes had similar levels of CREB and HSL phosphorylation (Fig 4E). Taken together, we conclude that the increased Ucp1 expression measured in whole adipose tissue is not likely due to an increased β-adrenergic receptor sensitivity in the adipocyte-specific *Arrdc3*-null mouse and may be a secondary and/or compensatory effect.

**Fig 5:**
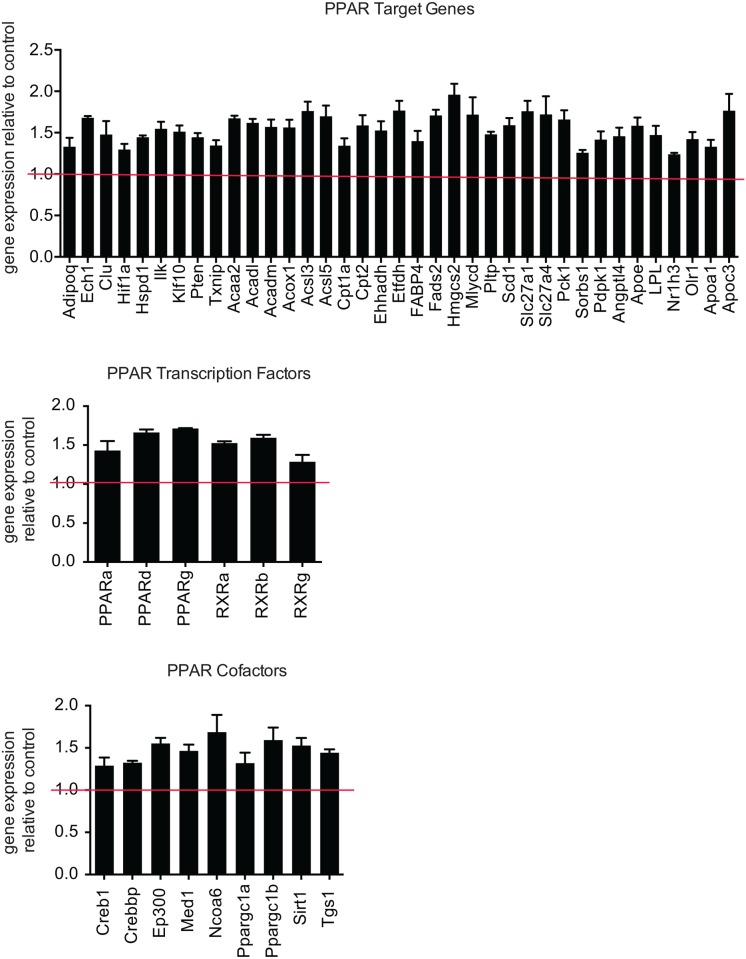
*Arrdc3* deletion increases PPAR target gene expression in adipocytes. Quantitative PCR array analysis of gene expression of PPAR target genes, PPAR cofactors and PPARs and associated transcription factors in control versus *Arrdc3* SVF-derived adipocytes *in vitro* (n = 3 mice per group). Only significantly different genes are displayed. *p≤ 0.05.

In addition to the β-adrenergic receptor signaling cascade, peroxisome proliferator-activated receptor (PPAR) activation participates in Ucp1 expression [15]. To test whether *Arrdc3*-null adipocytes had differences in PPAR pathways, we performed a quantitative PCR array of PPAR target genes and associated proteins on stromal vascular-derived control and *Arrdc3*-null adipocytes. *Arrdc3*-null cells had significantly increased expression of PPARα, δ and γ target genes (Fig 5). Additionally, PPAR cofactors and the PPARs themselves all had higher expression in the *Arrdc3*-null cells (Fig 5). These data suggest that the differences in Ucp1 expression in *Arrdc3*-null adipocytes could be due to changes in PPAR signaling, either through direct effects on the Ucp1 promoter or through effects on adipogenesis, as basal Ucp1 and Cidea tended to be higher in these cells (S4 Fig).

## Discussion

The primary focus of Arrdc3’s molecular role has been its interaction with the β2-adrenergic receptor, and there are now data from multiple laboratories that Arrdc3 is associated with the internalized β2-adrenergic receptor [6–11]. Because Arrdc3 has also been shown to interact with Nedd4 ubiquitin ligase [10], the prevailing hypothesis is that Arrdc3 regulates β2-adrenergic receptor recycling and, as a consequence, its signaling [6–11]. Arrdc3’s involvement in β2-adrenergic receptor action is a finding of particular significance as the β-adrenergic receptor is a principal activator and recruiter of brown/beige adipocytes [17] and has been the focus of multiple pharmacological obesity interventions [18].

We previously described Arrdc3 as being associated with human obesity and to be regulated by feeding/fasting in human adipose tissue [6]. The total *Arrdc3*-null mouse is resistant to weight gain, seemingly due to increased browning of white adipose tissue and an increased energy expenditure [6]. However, because adipose tissue browning and thermogenesis can be regulated by a variety of tissues [15], we generated an adipocyte-specific *Arrdc3*-null mouse to test whether adipocyte Arrdc3 has a role in white adipose tissue browning and energy expenditure.

Overall, adipocyte-specific deletion of *Arrdc3* resulted in a mild phenotype of decreased adiposity and a tendency towards increased energy expenditure. Therefore, we can conclude that there are other tissues in which Arrdc3 regulates energy expenditure and thermogenesis of adipose tissue, such as regions of the hypothalamus. Interestingly, although *Arrdc3* ablation in adipocytes had little to moderate effects on energy expenditure, these mice had dramatically increased expression of Ucp1 in white adipose tissue. Therefore, we conclude that adipocyte Arrdc3 participates in the regulation of Ucp1 expression but that other factors contribute to the metabolic role of Arrdc3.

Ucp1 expression is most commonly attributed to β-adrenergic receptor signaling [4]. As Arrdc3 has been shown to interact with the β2-adrenergic receptor *in vitro* [6–11], it was hypothesized that the increased Ucp1 expression in white adipose tissue of *Arrdc3*-null mice is due to increased signaling via the β2-adrenergic receptor. This hypothesis was supported by our previous data that white adipose tissue from *Arrdc3*-null mice responded to β-adrenergic receptor signaling with increased cAMP concentrations and increased lipolysis, relative to adipose tissue from control mice [6]. However, our previous experiments did not address the possible difference in the cellular composition of the adipose tissue. Here, we isolated the stromal vascular fraction from control and *Arrdc3*-null adipose tissue and differentiated them to adipocytes *in vitro*. In our model, *Arrdc3* ablation occurs upon expression of cre recombinase by the adiponectin promoter and we did not expect, nor observe, significant differences in the level of adipogenesis. Utilizing this *in vitro* system, we found that β-adrenergic receptor stimulation of *Arrdc3*-null adipocytes did not result in increased Ucp1 expression and, in fact, Ucp1 upregulation tended to be lower in the absence of Arrdc3.

We tested for differences in cAMP levels, PKA phosphorylation of CREB and HSL, and glycerol release/lipolysis between control and *Arrdc3*-null adipocytes upon β-agonism. The data presented here suggests that differences measured in Ucp1 expression are not a result of differences in canonical β-adrenergic receptor signaling. The β-adrenergic receptor has also been described to signal via MAPK and differences in this pathway could lead to the changes in Ucp1 expression (reviewed in [19]).

We also considered whether other regulators of Ucp1 expression could be affected by *Arrdc3* deletion. PPARs are known to interact with PGC1α at the Ucp1 promoter and upregulate expression [20]. Arrdc3 has been previously found to inhibit PPARγ activity [21] and therefore we measured the expression of PPAR target genes in our *Arrdc3*-null adipocytes. We found that the majority of PPAR target genes tested were upregulated in the *Arrdc3*-null adipocytes. This suggests that Arrdc3 regulates Ucp1 expression by modulating PPAR activity. Future experiments may determine if Arrdc3 regulation of PPAR leads to differences in the differentiation of the stromal vascular fraction to beige or white adipocytes, and consequently differences in gene expression and/or if PPAR is more active in adipocytes in the absence of Arrdc3.

Paradoxically, *Arrdc3* ablation *in vivo* resulted in increased Ucp1 expression, whereas ablation *in vitro* tended to decrease Ucp1 expression. We speculate that Arrdc3 has a positive role in the *in vivo* induction of Ucp1 and that *Arrdc3*-null animals may be remodeling their adipose depots during development to compensate for insufficient Ucp1 expression. This is supported by unpublished data from our lab that Ucp1 mRNA expression in subcutaneous adipose tissue of 15 day old adipocyte-specific *Arrdc3*-null pups is lower as compared to littermate controls. The concept of developmental compensation has been described by Schulz et al., where genetic ablation of type 1A bone morphogenetic protein receptor (Bmpr1A) resulted in impaired brown adipose tissue development and a subsequent browning of white adipose depots [22]. Further experimentation will be needed to explore this issue.

## Materials and methods

### Adipocyte-specific deletion of *Arrdc3*

All experiments were conducted in accordance with the Guide for the Use and Care of Laboratory Animals and approved by the Harvard Medical Standing Committee on Animals. Animals were euthanized by an overdose of isoflurane followed by cervical dislocation. *Arrdc3*fl/- mice were generated using a targeting vector developed by the Knock Out Mouse Program (KOMP), clone name PRPGS00100_B_H08. ES cells were electroporated with the targeting vector and after positive selection were injected into a donor blastocyst at the Genome Modification Facility of the Harvard Stem Cell Institute. Chimeras were selected and crossed with mice expressing Flp recombinase to remove the β-geo cassette. Mice were then bred to homozygocity for the floxed allele. Adipocyte-specific *Arrdc3* knockout mice were generated by breeding the *Arrdc3*fl/fl mice to a transgenic mouse expressing Cre recombinase under an adiponectin promoter (B6; FVB-Tg(Adipo-cre)1Evdr/J), Jackson Labs). Mice were fed a breeding diet (PicoLab Mouse Diet 20) with an energy content of 23% protein, 21% fat, and 55% carbohydrates and were housed in microisolator cages. All animals were housed at control temperature (20–21°C) and lighting (12 h dark/12 h light).

### Metabolic cage analysis

To assess multiple metabolic parameters, CLAMS (Columbus Instruments) metabolic cages was employed as previously described [23]. Briefly, mice were transferred to individual metabolic cages without bedding, with free access to food and tap water and acclimated for 48 h before starting measurements. Mice were subjected to non-invasive monitoring of gas exchange, physical activity and food intake and an Echo-MRI 3-in-1 Body composition analyzer (Echo Medical Systems) was employed to measure total body fat and lean mass. Respiratory exchange ratios (RER) were calculated as the ratio of carbon dioxide produced to oxygen consumed and energy expenditure (kcal/hour) were calculated from gas exchange. Values of energy expenditure were adjusted for lean body mass by ANCOVA. Physical activity was determined according to beam breaks within a grid of photosensors built into the cages. Total activity was defined as the total number of beam breaks. To assess food consumption and determine the cumulative amount of food eaten, a balance connected to each cage in the CLAMs apparatus was employed.

### Glucose homeostasis tests

For glucose tolerance tests (GTT) 6-week-old animals were fasted overnight and given 2 g/kg dextrose IP. For insulin tolerance tests (ITT), animals were fasted for 4 hours beginning in the morning and given 0.75 U/kg insulin (human, Sigma Aldrich) IP. Tail vein blood glucose was measured using a glucometer at preset time points and recorded. Glucose-stimulated insulin secretion was performed by fasting mice overnight, injecting 2 g/kg dextrose IP and collecting tail vein blood. Blood insulin concentrations were measured by ELISA (Mouse Ultrasensitive Isulin ELISA kit, ALPCO).

### Stromal Vascular Fraction (SVF) isolation, adipocyte differentiation and treatment

Subcutaneous adipose depots from mice were removed and digested in 1.5 U/ml collagenase type XI. The SVF pellet was resuspended and plated at 0.1x10^6^ cells/ml. Adipocyte differentiation was started after the cells became confluent. Differentiation reagents consisted of 1.72 μM insulin, 2 μM dexamethasone and 0.5 mM IBMX. Adipogenic media was replaced every other day. For isoproterenol treatment, cells were fasted for two hours in serum free media. Cells were treated with 10 μM isoproterenol or vehicle (H_2_O) for the specified length of time and then collected for further analysis. Cyclic AMP concentrations were measured using Enzo Direct cAMP ELISA kit. Glycerol concentrations were measured using Zenbio Cultured Human Adipocyte Lipolysis Assay kit.

### Quantitative RT-PCR

Total RNA was isolated from homogenized frozen tissues or from cultured cells using the RNeasy Mini kit (Qiagen). RNA was reverse transcribed using a High-Capacity cDNA Reverse Transcription Kit (Applied Biosystems). Gene expression was measured with Applied Biosystems reagents using a CFX384 Real-time System. The following TaqMan primer/probes were used; TBP (Mm00446971_m1), Arrdc3 (Mm00626887_m1), Ucp1 (Mm01244861_m1), Cidea (Mm00432554_m1), ATP synthase 6 (Mm03649417_g1), PRDM16 (Mm00712556_m1), PGC1α (Mm01208835_m1). PPAR target gene expression was performed using RT2 Profiler PCR Array Mouse PPAR Targets (Qiagen cat. no. 330231 PAMM-149ZA).

### Western analysis

Tissues were homogenized in RIPA buffer (150 mM NaCl, 0.1% Triton X-100, 0.5% sodium deoxycholate, 0.1% SDS, 50 mM Tris-HCl pH 8.0) supplemented with protease inhibitor (cOmplete Mini, Roche). The homogenate was centrifuged at 16,000 x g for 10 min at 4°C. The supernatant was assayed for protein concentration using a BCA assay. 15 μg of protein was resolved on a 10% SDS-PAGE gel (Invitrogen). Immunoblotting was performed using the following antibodies; P-CREB (Cell Signaling #9198), CREB (Cell Signaling #9197), pHSL (Cell Signaling #4139), HSL (Cell Signaling #4107), Ucp1 (Abcam #ab10983), and actin (Santa Cruz # sc-1615).

### Statistical analyses

Statistical significance was determined by a two-tailed Students t-test or a 2 way ANOVA with a significance value ≤0.05 using Prism Graphpad Software. Sample size refers to biological replicates.

## Supporting information

S1 Fig Increased expression of Ucp1 in visceral adipose tissue of adipocyte-specific *Arrdc3*-null mice relative to controls. Western analysis of Ucp1 protein expression in subcutaneous (SAT) and parametrial (VAT) adipose tissue.
(TIF)

S2 Fig Increased expression of Ucp1 in white adipose tissue of male adipocyte-specific *Arrdc3*-null mice. A) Adipocyte-specific *Arrdc3*-null male mice and littermate controls were weighed for 16 weeks and no differences in body weight were found (n = 4–17). B) Quantitative PCR analysis of gene expression in subcutaneous adipose tissue. (n = 3) *p<0.05. C) Western analysis of Ucp1 protein expression in subcutaneous adipose tissue.
(TIF)

S3 Fig Arrdc3 mRNA expression is lower in adipogenic treated SVF of adipocyte-specific *Arrdc3*-null mice. Quantitative PCR analysis of Arrdc3 gene expression in control versus adipocyte-specific *Arrdc3*-null cells after adipogenic treatment. (n = 4).
(TIF)

S4 Fig Basal beige adipocyte gene expression in control and *Arrdc3*-null adipocytes. Quantitative PCR analysis of gene expression in control versus adipocyte-specific *Arrdc3*-null cells. (n = 6,7).
(TIF)
