# Correction: Adipocyte arrestin domain-containing 3 protein (Arrdc3) regulates uncoupling protein 1 (Ucp1) expression in white adipose independently of canonical changes in β-adrenergic receptor signaling

**DOI:** 10.1371/journal.pone.0181492

**Published:** 2017-07-10

**Authors:** Shannon H. Carroll, Ellen Zhang, Bing F. Wang, Katherine B. LeClair, Arifeen Rahman, David E. Cohen, Jorge Plutzky, Parth Patwari, Richard T. Lee

Fig 1A is incorrect. Please see the entire correct Fig 1 here. Fig 2B is also incorrect. The western blot labeled “BAT” should read “VAT.” Please see the entire correct Fig 2 here.

**Fig 1:**
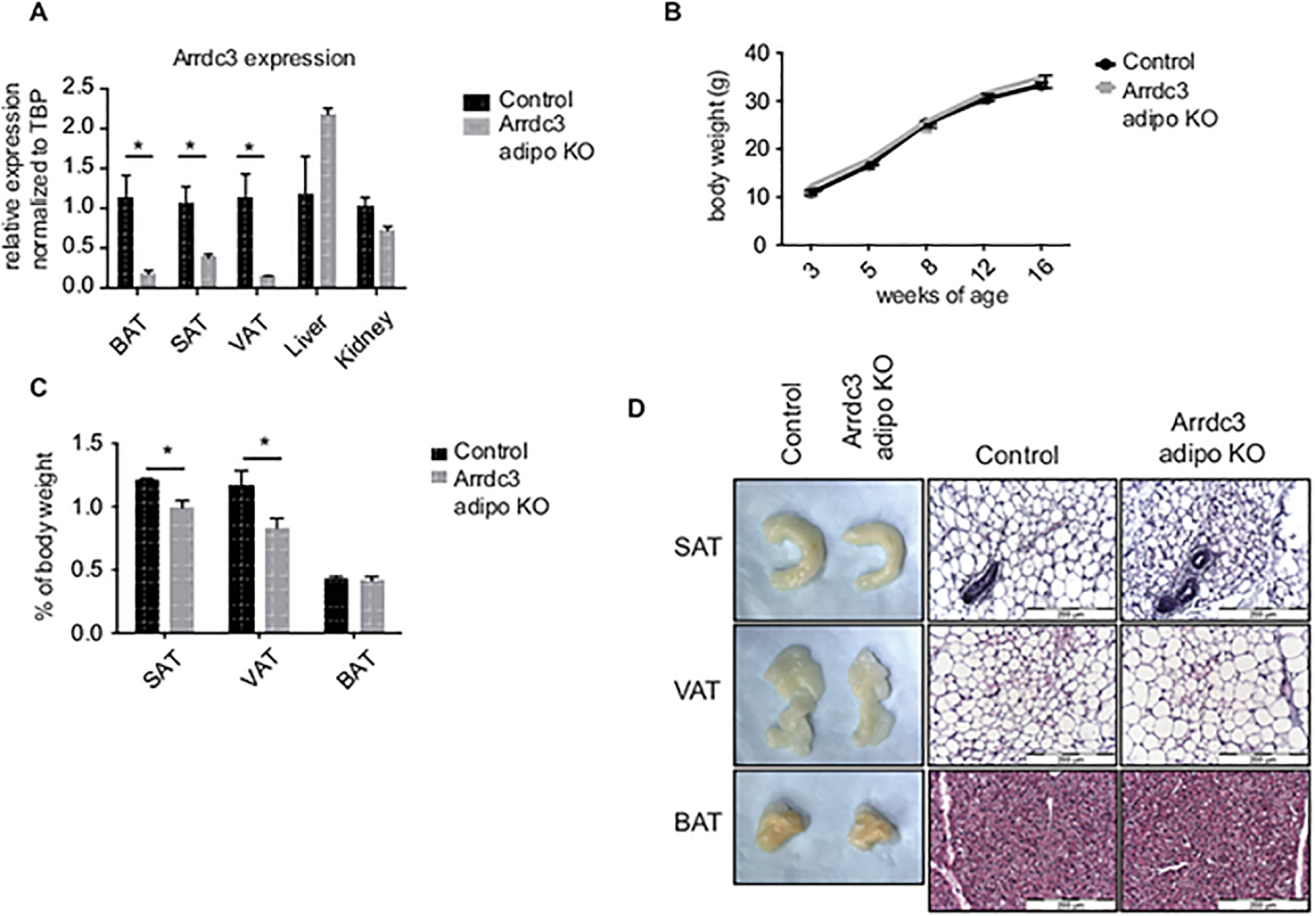
Characterization of adipocyte-specific *Arrdc3*-null mice. (A) To confirm adipocyte-specific deletion, Arrdc3 expression was measured in various tissues of Cre–(control) and Cre+ (*Arrdc3*-null) mice by quantitative PCR. Brown (BAT), parametrial (VAT) and subcutaneous adipose tissue (SAT) had significantly decreased *Arrdc3* expression while there was no significant difference in liver or kidney (n = 3–4). (B) Adipocyte-specific *Arrdc3*-null mice and littermate controls were weighed for 16 weeks and no differences in body weight were found (n = 4–10). (C) Specific adipose depots of female mice were weighed and normalized to total body weight. Subcutaneous (SAT) and parametrial (VAT) adipose tissue from adipocyte-specific *Arrdc3*-null mice weighed significantly less than controls (n = 5). (D) Representative macroscopic (formaldehyde fixed tissue) and microscopic appearance of subcutaneous (SAT), parametrial (VAT) and brown (BAT) adipose tissue from adipocyte-specific *Arrdc3*-null and control mice. Paraffin tissue sections were stained with hematoxylin and eosin and images were taken at 40x.

**Fig 2:**
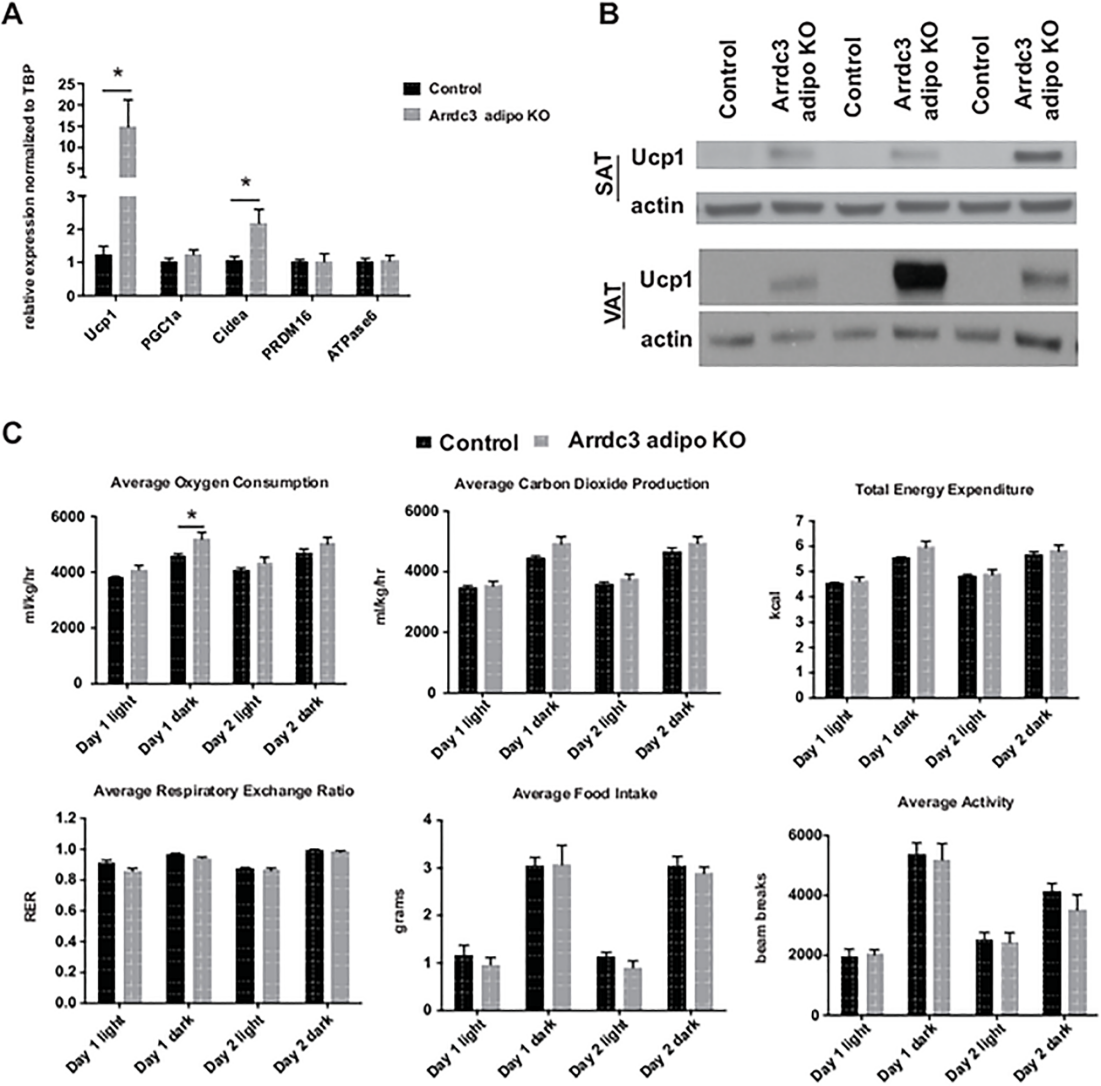
Increased expression of Ucp1 in white adipose tissue of adipocyte-specific *Arrdc3*-null mice. (A) Quantitative PCR analysis of gene expression in subcutaneous adipose tissue (n = 5–9). (B) Western analysis of Ucp1 protein expression in subcutaneous (SAT) and parametrial (VAT) adipose tissue. (C) 48 hours of CLAMS analysis of adipocyte-specific *Arrdc3*-null and control mice at 28°C ambient temperature (n = 5). *p≤ 0.05.
