# Supplementary figures and images for: Adipocyte arrestin domain-containing 3 protein (Arrdc3) regulates uncoupling protein 1 (Ucp1) expression in white adipose independently of canonical changes in β-adrenergic receptor signaling
Source: PLoS One. 2017 Mar 14;12(3):e0173823. doi: 10.1371/journal.pone.0173823 (PMC5349670; doi:10.1371/journal.pone.0173823)

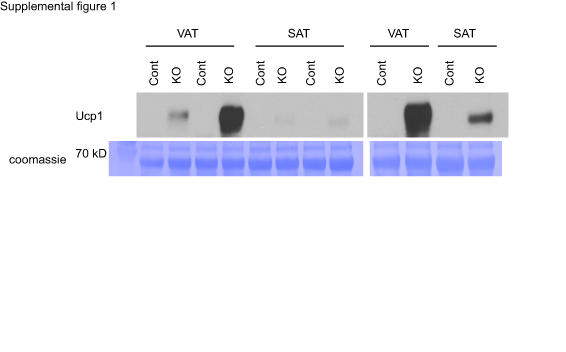

Supplement: S1 Fig — Western analysis of Ucp1 protein expression in subcutaneous (SAT) and parametrial (VAT) adipose tissue. (TIF) [file pone.0173823.s001.tif]

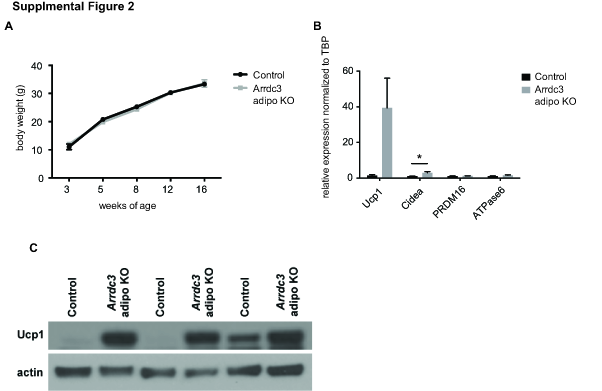

Supplement: S2 Fig — A) Adipocyte-specific Arrdc3-null male mice and littermate controls were weighed for 16 weeks and no differences in body weight were found (n = 4–17). B) Quantitative PCR analysis of gene expression in subcutaneous adipose tissue. (n = 3) *p<0.05. C) Western analysis of Ucp1 protein expression in subcutaneous adipose tissue. (TIF) [file pone.0173823.s002.tif]

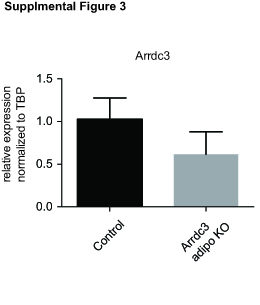

Supplement: S3 Fig — Quantitative PCR analysis of Arrdc3 gene expression in control versus adipocyte-specific Arrdc3-null cells after adipogenic treatment. (n = 4). (TIF) [file pone.0173823.s003.tif]

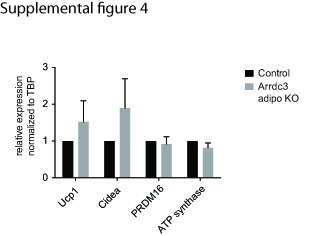

Supplement: S4 Fig — Quantitative PCR analysis of gene expression in control versus adipocyte-specific Arrdc3-null cells. (n = 6,7). (TIF) [file pone.0173823.s004.tif]
